# Therapeutic Contact Lenses with Polymeric Vehicles for Ocular Drug Delivery: A Review

**DOI:** 10.3390/ma11071125

**Published:** 2018-07-01

**Authors:** Seung Woo Choi, Jaeyun Kim

**Affiliations:** 1 Department of Health Sciences and Technology, Samsung Advanced Institute for Health Sciences & Technology (SAIHST), Sungkyunkwan University (SKKU), Suwon 16419, Korea; choizza@gmail.com; 2 School of Chemical Engineering, Sungkyunkwan University (SKKU), Suwon 16419, Korea; 3 Biomedical Institute for Convergence at SKKU (BICS), Sungkyunkwan University (SKKU), Suwon 16419, Korea

**Keywords:** contact lenses, ocular drug delivery, nanoparticles, polymeric vehicles, drug-eluting

## Abstract

The eye has many barriers with specific anatomies that make it difficult to deliver drugs to targeted ocular tissues, and topical administration using eye drops or ointments usually needs multiple instillations to maintain the drugs’ therapeutic concentration because of their low bioavailability. A drug-eluting contact lens is one of the more promising platforms for controllable ocular drug delivery, and, among various manufacturing methods for drug-eluting contact lenses, incorporation of novel polymeric vehicles with versatile features makes it possible to deliver the drugs in a sustained and extended manner. Using the diverse physicochemical properties of polymers for nanoparticles or implants that are selected according to the characteristics of drugs, enhancement of encapsulation efficiency and prolonged drug release are possible. Even though therapeutic contact lenses with polymeric vehicles allow us to achieve sustained ocular drug delivery, drug leaching during storage and distribution and the possibility of problems related to surface roughness due to the incorporated vehicles still need to be discussed before application in a real clinic. This review highlights the overall trends in methodology to develop therapeutic contact lenses with polymeric vehicles and discusses the limitations including comparison to cosmetically tinted soft contact lenses.

## 1. Introduction

The eye is an elaborate and complex organ equipped with various anatomical and physiological drug penetration barriers, including the precorneal and corneal barriers, the conjunctival barriers, the blood–aqueous barrier, and the blood-retinal barrier [1,2]. Because of these barriers, ocular drug delivery to the desired target tissue is a very challenging task for clinicians and scientists. There are various routes of administration for the treatment of eye diseases, including topical, intracameral, subconjunctival, subtenon, intravitreal, retrobulbar, and systemic routes [1,2]. Topical administration, including eye drops and ointments, is a non-invasive and commonly used administration route for anterior segment diseases, and accounts for more than 90% of ophthalmic formulations [3]. However, topically administered eye drops are rapidly washed out into the nasolacrimal duct due to the fast turnover rate and restoration time of the tear film and are eliminated by conjunctival blood and lymphatic flow [4]. For this reason, only 1–5% of the administered drug is absorbed into the target tissue [5] and the mathematically predicted bioavailability for drug delivery to the anterior chamber is less than 5% for lipophilic molecules and less than 0.5% for hydrophilic molecules [6]. To compensate for this low bioavailability, frequent administration of eye drops is usually needed, and this may cause poor patient compliance, particularly for chronic ocular diseases such as glaucoma and dry eye disease [7]. Moreover, from 18.2% to 80% of patients increase the risk of contaminating their eye drop bottle with facial microbes by touching their eye or face, and from 11.3% to 60.6% of patients do not administer exactly one drop when they apply the eye drops [8]. In other words, proper eye drop technique, which may affect the patients’ clinical outcomes, is not easy for most of the patients and may be a factor of the unexpected noncompliance [9].

**Figure 1:**
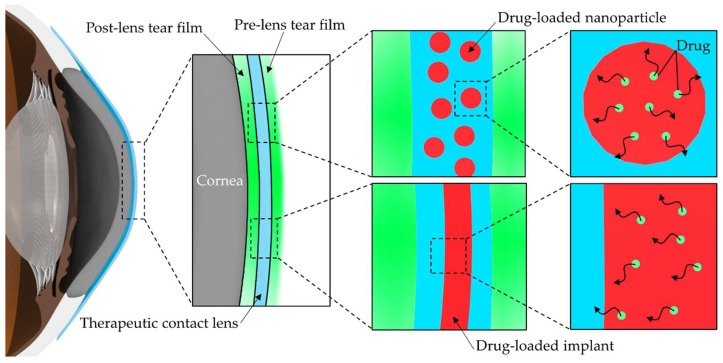
Schematic of drug release onto the ocular surface from drug-eluting therapeutic contact lens. Typical polymeric vehicles for therapeutic contact lenses include drug-loaded polymeric nanoparticles and drug-loaded polymeric implants inside contact lenses.

To remedy these drawbacks, drug-eluting or therapeutic contact lenses can be an excellent alternative for the treatment of eye diseases. Because drug delivery with therapeutic contact lenses increases the residence time of the drug in front of the cornea, the bioavailability of the drug can increase to approximately 50%, and improved bioavailability can enhance drug efficacy and minimize systemic side effects [7,10]. The platform of therapeutic contact lenses also may induce the patients’ high compliance due to the elimination of multiple drug administrations, especially for the patients wearing contact lenses for vision correction [11,12]. The first method developed to load drugs into contact lenses was by soaking contact lenses in drug solutions, but nearly all the drug in contact lenses was released within 1–3 h [13,14,15]. To overcome this drawback, many techniques to design therapeutic contact lenses have been developed, including a molecular imprinting technique, the entrapment of drug-loaded colloidal micro- and nanoparticles, drug-loaded implants in the contact lenses, sustained drug release using ionic interactions, drug delivery with vitamin E diffusion barriers, and supercritical fluid technology [16,17,18,19,20,21,22]. Among them, drug delivery using novel polymeric vehicles has been proposed to improve the controlled release of drugs with longer residence time on target tissues and is widely being studied in not only ocular drug delivery but also in targeted drug delivery to systemic circulation [23,24]. By definition, polymers, classified into natural and synthetic, are long-chain molecules with a high molecular weight and elaborately controlled polymeric structure, such as colloidal polymeric particles and implant, can elicit precise drug-releasing properties to the external environment [24,25]. Furthermore, polymeric materials exhibit versatility from a material point of view, and can be controllable in many aspects of their material properties, such as structure, configuration, and biomechanical behavior [23]. In general, polymer-based drug delivery has been based on drug diffusion from the polymeric matrix, erosion or biodegradation of polymers. Recently, responsive polymers by external stimuli, such as pH, temperature, etc., polymer-drug conjugates, polymeric encapsulation of drugs, and PEGylation for protein or drug carriers are broadly studied in polymeric drug delivery [4,23,24]. In the field of ophthalmology, there are many possibilities for polymeric drug delivery via periocular or intracameral injection, and subconjunctival or intravitreal implantation [1,4]. However, as the demand for non-invasive treatment increases, therapeutic contact lenses have received much attention recently. Since the entrapment of drugs in these vehicles makes additional partitions, the diffusion of drugs is considerably impeded and the drug release from these vehicles in therapeutic contact lenses can be sustained for a long period (Figure 1) [18,19]. This concept of additional partitioning in therapeutic contact lenses has proven its usefulness by mathematical models based on the numerically calculated solutions of Fick’s laws of diffusion, and the resistance to mass transport at the interface between drug-loaded vehicles and the matrix of contact lenses has been found to be an important factor in sustained drug release [26]. This review aims to provide the details from prior reports and look at recent trends for drug-loaded colloidal polymeric nanoparticles and implants in therapeutic contact lenses as polymeric vehicles for ocular drug delivery in the platform of contact lenses. Table 1 summarizes the characteristics of the various polymers as vehicles for therapeutic contact lenses discussed in this review for a better understanding in perspective.

## 2. Drug-Loaded Colloidal Polymeric Nanoparticles in Therapeutic Contact Lenses

Therapeutic contact lenses embedded with drug-loaded particles can be produced by incorporating various colloidal drug-encapsulated particles, such as polymeric micro- and nanoparticles, liposomes, microemulsions, and micelles [18,19,20,27,28,29,30,31,32]. Even though polymeric particles for embedding into contact lenses can be micro-sized, the essential features of contact lenses including optical and physical properties are easily affected by larger sized particles, especially in the micron range, so nanoparticles are mainly used in these days [33]. Polymeric nanoparticle-embedded contact lenses are usually fabricated in two steps: the synthesis of drug-loaded nanoparticles and the subsequent incorporation of nanoparticles into the matrix of contact lenses during the fabrication of contact lenses [18]. Generally, drug-loaded nanoparticles can be incorporated by mixing with the monomers of contact lenses before polymerization [29,30,31,33]. If the drugs are unstable in UV or heat required for the polymerization, these can be loaded into the already polymerized contact lenses by soaking in the particle solutions [31].

**Figure 2:**
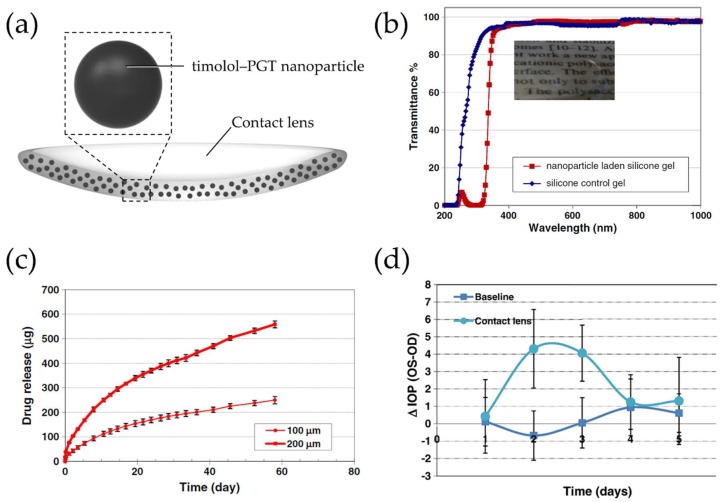
(**a**) Schematic of a therapeutic contact lens embedded with timolol–PGT nanoparticles; (**b**) Transmittance spectra of silicone control and PGT nanoparticle-embedded silicone hydrogel. The inset shows a photograph of the PGT nanoparticle-embedded silicone hydrogel; (**c**) Cumulative drug release profile from timolol–PGT nanoparticle-loaded hydrogels with 100 and 200 μm thickness, respectively; (**d**) Pharmacodynamic profile of timolol–PGT nanoparticle-loaded contact lenses in beagle dogs, which was expressed as the difference between the intraocular pressure (IOP) of the untreated (OS) and the treated eye (OD). The IOP-lowering effect due to timolol–PGT nanoparticle-loaded contact lenses was seen on day 2 and day 3. Reprinted with permission from [28].

Timolol maleate, a β-adrenergic receptor antagonist and an approved drug by the Food and Drug Administration (FDA) in 1979, is a very widely used anti-glaucoma drug and reduces intraocular pressure (IOP) by about 20–35% by decreasing aqueous humor formation [34,35,36,37,38]. It has high water solubility and can react with propoxylated glyceryl triacrylate (PGT) to form ester bonds [36,39]. Based on these properties, Jung et al. designed silicone hydrogel contact lenses embedded with timolol-loaded PGT nanoparticles (Figure 2a) [28]. The timolol-loaded PGT nanoparticles were synthesized by thermal polymerization of a mixture of PGT and timolol maleate. The drug-loaded PGT nanoparticles were then mixed with a precursor solution with bis-alpha,omega-(methacryloxypropyl) polydimethylsiloxane as a main component and the mixture was polymerized in a mold to obtain silicon hydrogel embedded with drug-loaded nanoparticles. Finally, the hydrogel was cut into circular pieces. Even after incorporation of timolol-loaded PGT nanoparticles into the silicone hydrogel contact lenses, it did not affect the contact lens’ properties including water content and transparency (Figure 2b). Moreover, nanoparticle-embedded contact lenses showed an extended timolol release at a constant rate for over 1 month (Figure 2c), which might be attributed to the hydrolysis of ester bonds between timolol and the PGT matrix. To examine the potential of this concept, Acuvue Oasys lenses, commercially acquired lenses, were soaked with drug-loaded nanoparticles and tested for IOP reduction in an animal model for glaucoma with Beagle dogs (Figure 2d). Preliminary studies showed therapeutic potential of these contact lenses by proving reduction of IOP in this animal model (Figure 2d).

**Figure 3:**
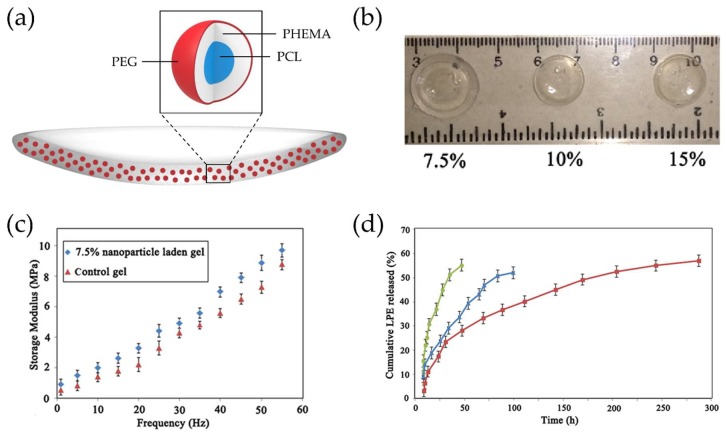
(**a**) Schematic of a therapeutic contact lens embedded with nanoparticles composed of an outer shell (polyethylene glycol), an inner shell (poly-hydroxyethylmethacrylate) and a core (polycaprolactone); (**b**) Photographs of transparent nanoparticle-loaded hydrogels with different nanoparticle loadings; (**c**) Frequency-dependent storage moduli of control hydrogel and nanoparticle-embedded hydrogel; (**d**) Cumulative drug release profiles from free loteprednol etabonate (LPE)-dispersed hydrogel (●), LPE-loaded nanoparticle (▲), and LPE-loaded nanoparticle-embedded hydrogel(■). Reprinted with permission from [29].

Unlike the case for hydrophilic drugs such as timolol, hydrophobic drugs are hard to load properly into contact lenses which have a water containing property. Loteprednol etabonate (LPE), an approved drug by the FDA in 1998, is an ester corticosteroid with high anti-inflammatory potency for the treatment of allergic conjunctivitis, uveitis, post-cataract surgical inflammation, etc., and has a low solubility in water (0.5 μg/mL) [40,41,42]. Polycaprolactone (PCL) is a hydrophobic biomaterial and FDA-approved bioresorbable polymer without toxic byproducts [43,44]. Nasr et al. produced hydroxyethylmethacrylate (HEMA)/*N*-vinylpyrrolidone (NVP) copolymer-based contact lenses embedding LPE-loaded PCL-based nanoparticles (Figure 3a) [29]. The PCL-based nanoparticles were prepared by surfactant-free miniemulsion polymerization (SFEP) and the resulting nanoparticles were composed of a hydrophilic outer shell (PEG; polyethylene glycol), a hydrophobic inner shell (poly-HEMA), and a hydrophobic core (PCL). Then poly(HEMA-co-NVP)-based hydrogels loaded with drug-loaded nanoparticles were prepared by free radical photopolymerization. The nanoparticle-loaded hydrogels exhibited high transmittance in the visible range at different nanoparticle loadings (Figure 3b) and comparable mechanical properties to typical commercial lenses (Figure 3c). These therapeutic contact lenses embedded with LPE-loaded nanoparticles exhibited an extended and sustained LPE release for about 12 days in in vitro experiments compared to both drug-loaded hydrogel and drug-loaded nanoparticles (Figure 3d). Because of the PCL-based nanoparticles as a vehicle, a better distribution of the drugs in poly-HEMA-based contact lenses was possible and therefore, the release time could be improved despite the use of hydrophobic drugs.

Dexamethasone (DMX), an anti-inflammatory and immunosuppressive drug, is another example of a hydrophobic glucocorticoid and has a very low loading efficiency by soaking method [45,46]. Behl et al. suggested a method that used ionic interactions between drugs and nanoparticles to load sufficient amounts of drug into the contact lenses [30]. They synthesized chitosan nanoparticles as a polymeric vehicle and loaded these into poly-HEMA contact lenses. Chitosan, an N-deacetylated derivative of chitin, is a cationic polysaccharide polymer and has good biocompatibility and biodegradability [47,48,49]. Because of chitosan’s positive charge, the negatively charged DMX can interact and be incorporated with it, and DMX-loaded chitosan nanoparticles did not exert a negative influence on the contact lens’ clarity. In vitro drug release experiments showed continuous DMX release over 22 days and 72% additional bioavailability of DMX in the form of drug-eluting contact lenses compared to an eye drop [30].

Prednisolone is also a widely used corticosteroid as an anti-inflammatory drug and has higher corneal permeability with a lower risk of IOP increase, even though glucocorticoid potency is lower than dexamethasone [50,51]. Moreover, prednisolone has a hydrophobic nature [52,53], so it needs a specific way to load into hydrophilic contact lenses. ElShaer et al. produced HEMA/methacrylic acid (MAA) copolymer-based contact lenses laden prednisolone-loaded poly(lactic-co-glycolic acid) (PLGA) nanoparticles [32]. As PLGA consists of relatively more hydrophilic glycolic acid content and relatively more hydrophobic lactic acid content, the property of the polymer is changed by varying the ratio between two components. It is also a biocompatible, biodegradable, and safely administrable polymer approved by the FDA [54,55]. The prednisolone-loaded PLGA nanoparticles were synthesized via an oil-in-water (O/W) microemulsion system. Contact lenses embedded with prednisolone-loaded PLGA nanoparticles exhibited similar mechanical properties to contact lenses without nanoparticles and good surface wettability. However, it was observed that the transparency of contact lenses decreased as the amount of embedded PLGA nanoparticles increased. An in vitro drug release profile showed slow and prolonged release of prednisolone showing 10.8% cumulative release over 24 h. This prolonged release of prednisolone was ascribed to the time-consuming process of breaking nanoparticles and the role of the matrix of contact lenses as a diffusion barrier [32].

Fungal keratitis is a leading cause of blindness especially in Asia and an intractable ocular infection requiring a prolonged course of therapy [56,57]. Natamycin, an approved antifungal drug by the FDA, is the drug of choice for treatment of fungal keratitis, but very frequent administration of natamycin eye drops is needed at hourly or two-hourly intervals over 1 week due to its poor penetrability across the cornea and being unable to get therapeutic concentrations by intravenous or subconjunctival injections [57,58,59]. Moreover, the loading of natamycin into therapeutic contact lenses is challenging because it has low water solubility and is light-sensitive [60]. Phan et al. suggested a solution for making natamycin-loaded therapeutic contact lenses by using poly(d,l-lactide)-dextran nanoparticles (Dex-b-PLA NPs) as a vehicle [31]. Natamycin-loaded Dex-b-PLA NPs were synthesized by nanoprecipitation. The core-shell structured nanoparticles were produced consisting of a hydrophobic PLA core and a hydrophilic dextran outer shell [61]. To avoid decomposition of natamycin during photo-crosslinking in the lens formation, natamycin-loaded Dex-b-PLA NPs were incorporated in the preformed contact lenses by soaking the lenses for 7 days in the solution of Dex-b-PLA NPs after polymerization of contact lens precursors such as poly-HEMA and *N*,*N*-dimethyl acrylamide. The loading amount in the contact lenses was increased in the case of natamycin-loaded Dex-b-PLA NPs compared to natamycin-dissolved solution. Additionally, an in vitro drug release profile showed a reduced burst release by 21–54% for natamycin-loaded Dex-b-PLA NPs-embedded contact lens materials, and extended release for up to 12 h, compared to 1 h by conventional drug loading methods [31]. This study showed the superior loading potential of drug-loaded polymeric nanoparticles into contact lenses despite incorporation between particles and contact lenses by a simple soaking method.

## 3. Drug-Eluting Polymeric Implants in Therapeutic Contact Lenses

Although the incorporation of drug-loaded nanoparticles into contact lenses shows its therapeutic potential by successfully demonstrating the sustained release of a loaded drug, there is always the possibility of changing intrinsic contact lens properties such as optical transparency, mechanical property and oxygen permeability because of the distribution of nanoparticles throughout the whole contact lens matrix [62]. To prolong the release of drugs and increase the drug-loading capacity without affecting the intrinsic properties of contact lenses, many researchers have focused on the development of drug-eluting polymeric devices inside contact lenses in the form of implants or films with clear central zones [62,63,64,65,66,67]. As the average of normal pupil diameter in adults ranges from 2 to 4 mm in bright light and 4 to 8 mm in the dark, a transparent central zone above 5 mm in diameter would be enough for clear visual function [68].

**Figure 4:**
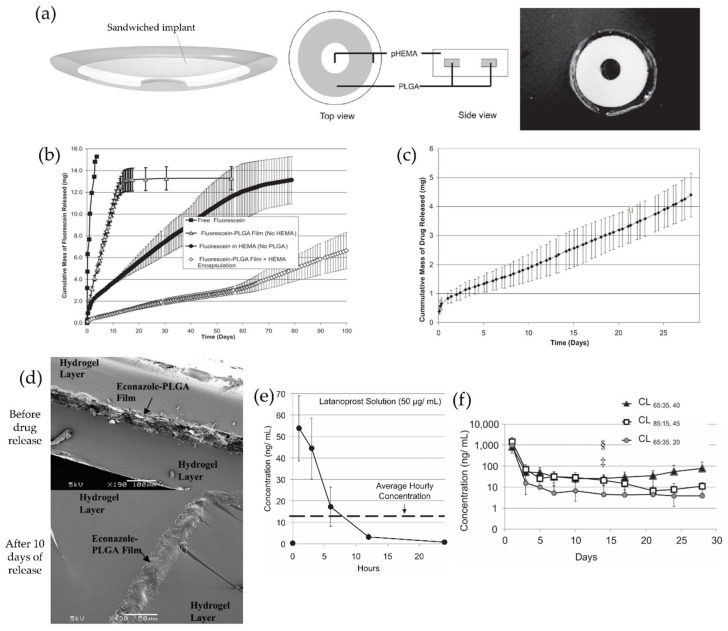
(**a**) Schematic of a therapeutic contact lens embedded with sandwiched polymeric implant (left, middle) and photograph (right) of a ciprofloxacin-loaded PLGA implant-embedded poly-HEMA-contact lens with a 5-mm clear central zone; (**b**) Cumulative drug release profiles for 100 days from free fluorescein powder, fluorescein-PLGA films, fluorescein-coated poly-HEMA-contact lens and poly-HEMA-contact lens embedded with fluorescein-PLGA films; (**c**) Ciprofloxacin release from PLGA implant-embedded contact lenses in vitro. Reprinted with permission from [63]; (**d**) SEM images (X190) of econazole-PLGA films within prototype contact lenses made of poly-HEMA hydrogel in before drug release (up) and in after 10 days of release (down). Reprinted with permission from [64]; (**e**) Concentration of latanoprost in aqueous humor after topical instillation of latanoprost solution (50 μg/mL); (**f**) Concentrations of latanoprost in aqueous humor in rabbits wearing latanoprost-eluting contact lenses. Reprinted with permission from [65].

Ciolino et al. reported the efficacy of therapeutic contact lenses containing PLGA films impregnated with several types of drugs, including ciprofloxacin, econazole, and latanoprost [63,64,65]. Ciprofloxacin, a second-generation fluoroquinolone, is one of the most commonly used broad-spectrum antibiotics and it exhibits antibacterial effects by inhibiting gyrase and topoisomerase IV, essential enzymes for bacterial cell growth and division [69,70,71]. However, it showed precipitate formation on the surface of contact lenses soaked in drug solution due to its poor solubility in water [72,73,74,75,76]. The ciprofloxacin-loaded PLGA films were prepared by a solvent casting method and these were punched out as a 14-mm outer diameter ring with a 5-mm central aperture for high central transmittance. Then these films were incorporated inside poly-HEMA contact lens, such as a sandwich (Figure 4a) [63]. The thickness of ciprofloxacin-loaded PLGA films and overall thickness of contact lenses were quite thick, about 225 μm and 450 μm, respectively. The release profiles with fluorescein as a preliminary study showed continuous and sustained release of fluorescein from a poly-HEMA-contact lens embedded with fluorescein-PLGA films for at least 100 days (Figure 4b). In vitro ciprofloxacin release profiles showed a similar sustained release with zero-order kinetics over 4 weeks after small initial burst release (Figure 4c). Additionally, the drug release could be manipulated by either adjusting the ratio of drug to PLGA film or changing PLGA’s molecular weight.

Econazole is a low cost, broad-spectrum antifungal drug in the azole class and has comparable therapeutic efficacy as natamycin for filamentary fungal infections [77,78,79]. However, it has very poor water solubility, and thus, it requires a specific method to load ophthalmic formulations [80,81]. Ciolino et al. have tried to solve these problems by designing antifungal contact lenses with embedded econazole-loaded PLGA film inside [64]. Econazole was successfully loaded into PLGA film consisting of 65% lactic acid and 35% glycolic acid with high molecular mass (118 kDa), and this film was sandwiched between two poly-HEMA layers (Figure 4d). The thickness of drug-loaded PLGA film and drug-loaded PLGA film-containing contact lens in dry state were still too thick, about 150 μm and 450 μm, respectively, to apply comfortably on the eye surface. Despite this drawback, this prototype of antifungal contact lenses exhibited good fungicidal effects for at least 3 weeks in co-cultivation of a *Candida albicans* suspension and the contact lenses [64].

Latanoprost, a prostaglandin F2α analog, is a first-line drug of choice for treatment of glaucoma and has an IOP-lowering effect by enhancing uveoscleral outflow [82,83]. It is also challenging for ocular topical administration due to its poor solubility in water [82,83]. Based on previous research, Ciolino et al. went one step further and demonstrated achieving therapeutic concentrations inside the anterior chamber of the eye with contact lenses embedded with latanoprost-loaded PLGA film in an animal study [65]. Latanoprost-loaded PLGA-films of 20, 40, and 45 μm thickness were produced and embedded into the polymer-based contact lenses in a sandwich-manner. The central thickness of the contact lenses after film embedding was about 300 μm in dry state. In vivo drug release experiments were conducted on New Zealand white rabbits wearing single contact lenses continuously for one month. No serious side effects were observed and the comparable latanoprost concentrations in the aqueous humor were achieved for at least one month by therapeutic contact lenses, compared to those achieved with a commercial solution of latanoprost (Figure 4e,f). Despite the disadvantage of the high thickness of these contact lenses, Ciolino et al. significantly reduced the thickness of latanoprost-loaded PLGA films and proved the safety and drug delivery efficiency to the anterior chamber of these therapeutic contact lenses [65].

**Figure 5:**
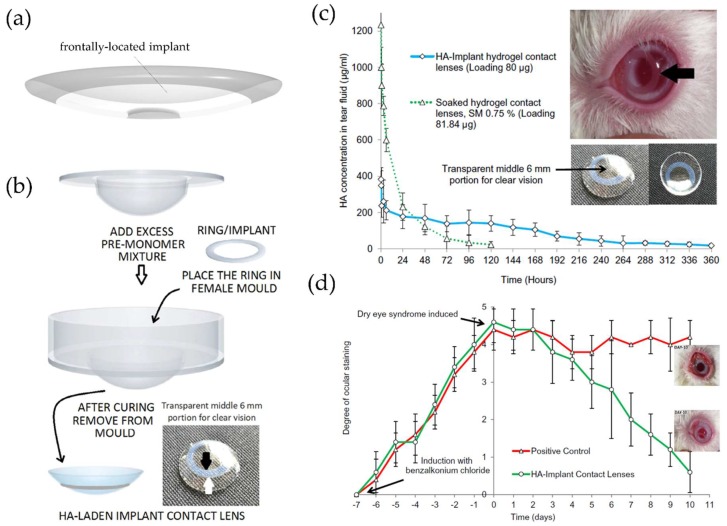
(**a**) Schematic of therapeutic contact lenses embedded with frontally-located implant; (**b**) Schematic of the fabricating process of contact lenses embedded with an HA-laden implant (Blank arrow: inner margin of the implant, white arrow: outer margin of the implant); (**c**) hyaluronic acid (HA) release profile in tear fluid after wearing sterilized HA implant contact lenses and soaked contact lenses on rabbit eyes; (**d**) changes of ocular staining levels in dry eye disease-induced rabbit eyes by benzalkonium chloride treated with HA-implant contact lenses. Reprinted with permission from [66].

It is possible to make implants for drug release with the same polymeric material used in contact lenses [62,66]. Hyaluronic acid (HA) is a very hydrophilic natural polymer consisting of repeating disaccharide units of *N*-acetyl-d-glucosamine and d-glucuronic acid [84,85,86]. It stimulates corneal epithelial proliferation and migration and has been used for treatment of dry eye disease [87,88,89]. Even though HA exhibited a relatively prolonged ocular surface residence time compared to other artificial tears [90], extended residence time is needed for pathologic conditions of the ocular surface such as a severe form of dry eye disease and Sjögren syndrome. Maulvi et al. designed a frontally-located HA-loaded poly-HEMA implant onto the poly-HEMA lens matrix (Figure 5a) [66]. The HA-loaded poly-HEMA implant was first synthesized by mixing HA with the HEMA/MAA monomer mixture and subsequently photo-polymerized. After placing the prepared implant in a female mold, excess pre-monomer mixture was added, and UV polymerization was done with placing the male mold in position. As a result, the prepared implant was incorporated into poly-HEMA contact lenses at the front surficial region (Figure 5b). Poly-HEMA is a hydrophilic hydrogel with high water content and has been widely used in biomedical applications, including contact lenses and ophthalmic drug delivery systems, due to its safety and excellent biocompatibility [91,92]. MAA is a hydrophilic co-monomer which can be polymerized with HEMA and is used to enhance water content because of the properties of being ionized above pH 5.5 and imparting a negative charge to hydrogels [93,94,95]. In vivo HA release experiments using rabbits exhibited prolonged HA release over 15 days with about an 80 μm-thick HA-loaded implant and 100 μm-thick contact lenses, compared to release within 120 h for HA-soaked contact lenses (Figure 5c). Moreover, in vivo efficacy studies in benzalkonium chloride-induced dry eye disease rabbits by evaluating ocular surface damage according to the degree of corneal fluorescein staining showed the enhanced healing effects of contact lenses with HA-loaded implants (Figure 5d) [66]. Alvarez-Lorenzo et al. also reported that incorporation of MAA into poly-HEMA hydrogels improved the timolol-loading capacity [94], while Weeks et al. reported that incorporation of higher concentrations of MAA reduced the amounts of HA loading in poly-HEMA hydrogels [95], which is probably due to the negatively charged HA with a pKa 3~4 in physiological conditions [96,97]. Therefore, it may be necessary to adjust each component in contact lenses to optimize HA loading.

**Figure 6:**
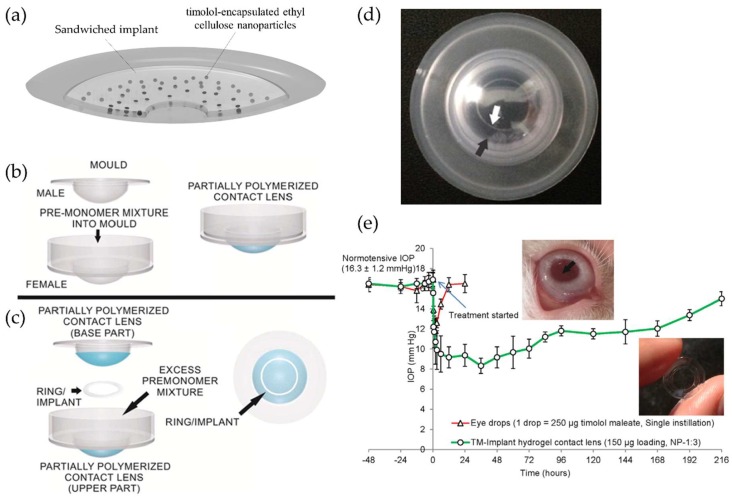
(**a**) Schematic of therapeutic contact lenses embedded with a sandwiched polymeric implant with timolol-encapsulated ethyl cellulose nanoparticles; schematic showing the process of fabricating contact lenses by (**b**) fabrication of partially UV-polymerized contact lenses and then (**c**) implantation of a ring implant between partially polymerized contact lenses; (**d**) Photograph of implant-embedded contact lens on a male mold showing a clear 6 mm central aperture and a translucent ring (White arrow: inner margin of the implant, black arrow: outer margin of the implant); (**e**) Change in intraocular pressure (IOP) in rabbits treated with eye drop and timolol-laden implant-embedded contact lens. Reprinted with permission from [62].

It is also possible to improve the drug-loading capacity without deteriorating the intrinsic optical and physical properties of contact lenses by combining two concepts of polymeric nanoparticles and implants in the contact lenses. Maulvi et al. reported the incorporating effects of timolol-encapsulated ethyl cellulose (EC) nanoparticles inside HEMA/MAA implants for controlled ocular drug delivery (Figure 6a) [62]. EC, one of the widely cellulose derivatives in the pharmaceutical field, is an ethyl ether of cellulose and a hydrophobic, biocompatible, non-biodegradable polymer with safety [98,99,100,101,102]. Timolol-encapsulated EC nanoparticles were incorporated into HEMA/MAA ring-shaped implants and then these implants were sandwiched inside poly-HEMA contact lenses. The nanoparticle-embedded implant was placed along with excess of pre-monomer mixture between the female mold and the male mold, both holding partially polymerized contact lens (Figure 6b,c), and then UV polymerization was done to produce the proto-type contact lenses embedded with a polymeric implant loaded with drug nanoparticles (Figure 6d). The resulting therapeutic contact lenses exhibited extended release and an IOP-lowering effect for 192 h in rabbits without significant ocular complications (Figure 6e). All components in therapeutic contact lenses, such as the hydrogel matrix of contact lenses, the ring-shaped implant, and the EC nanoparticles, acted as multilayer diffusion barriers, and thus could contribute to significant lowering of the release rate of the drug.

The multilayer contact lenses composed of drug-loaded hydrogel glue placed between two contact lenses were also developed. Hyatt et al. produced therapeutic contact lenses with vancomycin- or gentamicin-loaded fibrin hydrogels [67]. Gentamicin is a widely used aminoglycoside antibiotic against Gram-positive and Gram-negative bacteria especially *Pseudomonas aeruginosa*, and vancomycin is an effective antibiotic for Gram-positive bacteria especially antibiotic-resistant bacteria including methicillin-resistant *Staphylococcus aureus* [103,104,105]. Fibrin, a protein-based natural biopolymer, is polymerized into the form of hydrogels from fibrinogen activated by the protease thrombin and can be biodegraded by plasmin-mediated fibrinolysis [106,107,108]. The fibrin gels loaded with vancomycin or gentamicin were prepared and fixed by fibrin glue between two conventional contact lenses. These antibiotic-loaded fibrin gel-encapsulated contact lenses exhibited in vitro extended release and good bactericidal effects. Although the thickness of these contact lenses is too thick to apply on the eyes and the opacity of fibrin gels reduces the optical transmittance of contact lenses, the proof-of-concept of the therapeutic potential of the multilayer lenses in ocular drug delivery was demonstrated. As the authors have noted, the optical clarity of contact lenses for antibiotic delivery may be not necessary because visual impairment has already occurred in the patients with severe ocular infections [67]. However, a deformed, discolored cornea with scars or opacities can deteriorate the self-confidence of patients and affect the social and professional lives of patients negatively [109]. Therefore, the adjustment of implants’ properties including color, shape and location inside contact lenses may be needed.

## 4. Current Limitations and Novel Approaches to Improve the Properties of Therapeutic Contact Lenses Including Drug Release

Polymeric vehicles including nanoparticles and implants for embedding into therapeutic contact lenses can increase the partitioning of drugs and prevent the interaction between drug molecules and the polymerization mixture, so it can endow additional diffusion resistance to drugs for extended controlled delivery [18,19]. In most of the studies described above, these platforms exhibit reduced burst release and prolonged release, and the therapeutic efficacy of these was sufficiently demonstrated by in vitro and in vivo experiments. However, there are also several downsides to these platforms that needs to be considered in the development of therapeutic contact lenses for the practical use.

**Figure 7:**
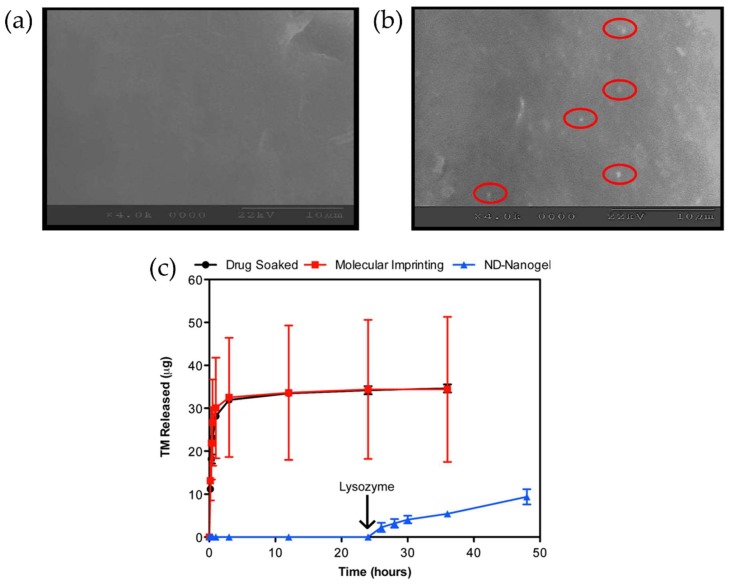
SEM images of (**a**) the surface of pure poly-HEMA contact lens; (**b**) the surface of nanoparticle-laden poly-HEMA contact lens with visible nanoparticles (red circle). Reprinted with permission from [30]; (**c**) Timolol-eluting profiles from drug-soaked (black line), molecularly imprinted (red line) and nanodiamond nanogel-embedded contact lenses (blue line), showing lysozyme-triggered drug release. Reprinted with permission from [125].

Firstly, the surface roughness of contact lenses needs to be more characterized and improved when new designs of therapeutic contact lenses are developed. Surface roughness of contact lenses is known as an important factor for bacterial transfer from contact lenses and deposit formation composed of tear proteins on the surface, and affects bacterial adhesion to surfaces, especially for initial adhesion [110,111,112,113]. Moreover, nanoscale surface roughness may have a greater impact on bacterial adhesion [114]. In contact lenses embedded with nanoparticles, nanoparticles could be located on the surface of contact lenses and this could increase the surface roughness of contact lenses [30] (Figure 7a,b). Therefore, there is a possibility that nanoparticles on the surface of contact lenses may influence bacterial adhesion which may have an adverse effect on eye irritation and infection. Additionally, polymeric vehicles such as nanoparticles are generally not covalently bonded to any part of the polymer matrix of contact lenses, so escaped vehicles themselves can cause ocular irritation [17].

In the same vein as nanoparticles, drug-loaded implants may exert the same effect on the surface of contact lenses. Most of these implants are embedded in a sandwiched manner or placed on the surface of contact lenses, and these are similar to the structure of cosmetically tinted soft contact lens. A tinted soft contact lens is composed of a specially designed pigmented peripheral area by decorating the iris parts for aesthetic effects, and a pigment-free transparent central optical zone [115,116,117]. It can be categorized by the location of pigments, an enclosed pigment layer inside the matrix of the contact lens without surface exposure or a pigment located on the surface in direct contact with the cornea or palpebral conjunctiva [115,118]. In case of tinted soft contact lenses with pigments on the surface, the pigmented areas showed significantly higher surface roughness compared to the pigment-free area [117,118]. Even though the surface roughness at pigmented areas was lowered in the case of tinted soft contact lenses with enclosed pigments inside contact lenses compared to those with pigments on the surface, the ocular surface status and the subjective symptom score of tinted soft contact lenses were inferior compared to conventional clear contact lens without pigments, regardless of the location of pigments [115]. Moreover, higher bacterial adhesion was observed in tinted soft contact lenses due to increased surface roughness, especially in case of those with pigments on the surface [119,120]. Based on the lessons from tinted lenses, drug-loaded implants on the surface or inside contact lenses may exhibit similar effects in the aspect of bacterial adhesion due to increased surface roughness. In addition, the cases of tinted soft lenses in which the pigment is located on the surface could be applied to investigate the effects of nanoparticles on the surface of contact lenses. Although the size varies from about 100 nm to 110 μm depending on the kind of pigment particles, it seems similar to the aspect of the size of drug-loaded nanoparticles [121]. Moreover, there was a case report about the occurrence of corneal damage by direct contact with exposed pigments of cosmetically tinted soft contact lens [122]. Additional studies are needed to determine to what effect nanoparticles or implants on the surface of contact lenses may have on bacterial adhesion and clinical manifestation.

A second important issue that needs to be considered is the loss of the drug loaded in contact lenses during storage and distribution. To use drug-loaded therapeutic contact lenses in real markets, the leaching or loss of the drugs from the contact lenses during storage and distribution should be avoided or minimized. Jung et al. showed that drug-loaded contact lens hydrogels could release drugs at therapeutic ranges for 20 days after 5 months packaging in a refrigerator at 4 °C, representing that refrigerated conditions are required to diminish the drug loss in packaging [28]. Ciolino et al. proposed the use of additional drugs in the packaging medium for the equilibrium between the drugs in the packaging medium and the drugs in contact lenses to delay drug leaching [63]. However, these two solutions may be impractical from an economic point of view, so manipulating the properties of polymeric vehicles rather than changing conditions of storage and distribution would be practical solutions. For example, specialized polymer vehicles that degrade under certain conditions could be one of the solutions for controlled ocular drug delivery without drug leaching in the process of storage and distribution. Chitosan is an *N*-deacetylated derivative of chitin, but chitosan also contains less than 40% *N*-acetyl-d-glucosamine residues [123]. Lysozyme, one of the enzymes in tears, can hydrolyze the β(1–4) linked *N*-acetyl-d-glucosamine and d-glucosamine in chitosan [124]. Using chitosan’s degradability by lysozyme, a few studies produced therapeutic contact lenses embedded with polymeric vehicles composed of chitosan and extended, controlled drug release from therapeutic contact lenses was observed only if lysozyme was added (Figure 7c) [125,126]. The rate of degradation by lysozyme was also controllable by adjusting the degree of acetylation of chitosan [125]. Similarly, controlled drug release can be achieved using a pH-sensitive polymer. Eudragit S-100, a pH-sensitive anionic copolymer consisting of methacrylic acid and methyl methacrylate is dissolved when the condition is above pH 7.0 [127,128]. Using drug-loaded nanoparticles made by Eudragit S-100, in vitro and in vivo drug release profiles exhibited a low initial burst and sustained the drug release, and the negligible leaching of drugs was observed in packaging solution with pH 6.5 PBS for 3 months [128]. Of course, careful inspection is needed in this concept with respect to surface and property changes due to degradation of the nanoparticles in contact lenses.

## 5. Conclusions and Perspectives

Polymers can be described as prospective vehicles for drug delivery because of their versatile properties including biocompatibility, bioavailability, and good mechanical properties. However, unique manipulation or modification of polymeric vehicles is needed for ocular drug delivery due to the unusual anatomical structure of the eyes, such as multiple barriers against drug penetration or a rapid wash-out system to shorten the residence time of drugs. Moreover, polymeric vehicles should be selected appropriate to the characteristics of drugs, such as hydrophobicity and solubility. Therefore, proper drug delivery to ocular tissues is challenging. Recent developments in ocular drug delivery using polymeric vehicles and the platforms of contact lenses allow us to achieve better results in the aspect of sustained and extended drug release profiles. Various drugs have been loaded into contact lenses with polymeric vehicles properly according to the physicochemical properties of drugs, and sustained release in the therapeutic range of drugs, even the therapeutic effect, has been confirmed by a variety of test equipment in vitro and in vivo. However, there are still considerations to improve therapeutic contact lenses embedded with polymeric vehicles in the clinic.

In this review, we highlighted the properties of polymers and drugs that are loaded on contact lenses. Understanding the relationship between polymeric vehicles and the loaded drug, or between polymeric vehicles and polymers of the matrix of contact lenses, will be the fundamental basis in designing efficient therapeutic contact lenses. Moreover, the goals of these contact lenses should aim for application in the clinic. For this, the safety issue that originates from changing surface roughness due to polymeric vehicles including nanoparticles and implants, and drug loss issue during the process of storage and distribution, should be resolved. The thinner and more transparent drug-loaded implant corresponding to the entire area of the lens without a central optical zone and the development of new polymeric material which reacts with the surrounding environment for controllable drug delivery would be another alternative to solve these issues. Alternatively, a paradigm shift it may be needed for therapeutic contact lenses to be applied practically to the real world.

## Figures and Tables

**Table 1:** Characteristics of various polymers as vehicles for therapeutic contact lenses.

Polymer	Characteristics	Ref.
Propoxylated glyceryl triacrylate (PGT)	Polymer having multiple vinyl functionalities	[28,39]
Polycaprolactone (PCL)	Hydrophobic and FDA-approved bioresorbable polymer without toxic byproducts	[43,44]
Chitosan	Cationic polysaccharide polymer with good biocompatibility and biodegradability including lysozyme-related degradability	[47,48,49,124,125,126]
poly-(lactic-co-glycolic acid) (PLGA)	Biocompatible, biodegradable and FDA-approved polymer that can change properties by varying the ratio of glycolic acid to lactic acid	[54,55]
Poly (d,l-lactide)-dextran (Dex-b-PLA)	Core-shell structured nanoparticles consisting of PLA core and dextran outer shell	[31,61]
Poly-HEMA ^1^	Hydrophilic hydrogel with high water content having excellent biocompatibility	[91,92]
Ethyl cellulose (EC)	Hydrophobic, biocompatible, non-biodegradable polymer	[98,99,100,101,102]
Fibrin	Protein-based natural biopolymer having biodegradability by plasmin-mediated fibrinolysis	[106,107,108]
Eudragit S-100	pH-sensitive anionic copolymer having dissolving property in above pH 7.0	[127,128]

^1^ Hydroxyethylmethacrylate.
